# Rotavirus genotypes in children under five years hospitalized with diarrhea in low and middle-income countries: Results from the WHO-coordinated Global Rotavirus Surveillance Network

**DOI:** 10.1371/journal.pgph.0001358

**Published:** 2023-11-28

**Authors:** Sebastien Antoni, Tomoka Nakamura, Adam L. Cohen, Jason M. Mwenda, Goitom Weldegebriel, Joseph N. M. Biey, Keith Shaba, Gloria Rey-Benito, Lucia Helena de Oliveira, Maria Tereza da Costa Oliveira, Claudia Ortiz, Amany Ghoniem, Kamal Fahmy, Hossam A. Ashmony, Dovile Videbaek, Danni Daniels, Roberta Pastore, Simarjit Singh, Emmanuel Tondo, Jayantha B. L. Liyanage, Mohammed Sharifuzzaman, Varja Grabovac, Nyambat Batmunkh, Josephine Logronio, George Armah, Francis E. Dennis, Mapaseka Seheri, Nonkululeko Magagula, Jeffrey Mphahlele, Jose Paulo G. Leite, Irene T. Araujo, Tulio M. Fumian, Hanan EL Mohammady, Galina Semeiko, Elena Samoilovich, Sidhartha Giri, Gagandeep Kang, Sarah Thomas, Julie Bines, Carl D. Kirkwood, Na Liu, Deog-Yong Lee, Mirren Iturriza-Gomara, Nicola Anne Page, Mathew D. Esona, M. Leanne Ward, Courtnee N. Wright, Slavica Mijatovic-Rustempasic, Jacqueline E. Tate, Umesh D. Parashar, Jon Gentsch, Michael D. Bowen, Fatima Serhan

**Affiliations:** 1 Department of Immunization, Vaccines and Biologicals, World Health Organization Headquarters, Geneva, Switzerland; 2 Department of Infectious Disease Epidemiology, London School of Hygiene and Tropical Medicine, London, United Kingdom; 3 School of Tropical Medicine and Global Health, Nagasaki University, Nagasaki, Japan; 4 World Health Organization, Regional Office for Africa, Brazzaville, Congo; 5 World Health Organization, Inter Country Support Team, Harare, Zimbabwe; 6 World Health Organization, Inter Country Support Team, Ouagadougou, Burkina Faso; 7 Pan American Health Organization, World Health Organization, Washington District of Columbia, Washington, DC, United States of America; 8 World Health Organization, Regional Office for the Eastern Mediterranean, Cairo, Egypt; 9 World Health Organization, Regional Office for Europe, Copenhagen, Denmark; 10 World Health Organization, Regional Office for South East Asia, Delhi, India; 11 World Health Organization, Regional Office for the Western Pacific, Manila, Philippines; 12 Noguchi Memorial Institute for Medical Research, University of Ghana, Accra, Ghana; 13 World Health Organization Regional Reference Laboratory for Rotavirus, Diarrhoeal Pathogens Research Unit, Department of Virology, Sefako Makgatho Health Sciences University, Pretoria, South Africa; 14 Laboratory of Comparative and Environmental Virology, Oswaldo Cruz Institute, Fiocruz, Rio de Janeiro, Brazil; 15 Bacterial and Parasitic Diseases Research Program, U.S. Naval Medical Research Unit-3, Cairo, Egypt; 16 Republican Research and Practical Center for Epidemiology and Microbiology, Minsk, Belarus; 17 Division of Gastrointestinal Sciences, The Wellcome Trust Research Laboratory, Christian Medical College, Vellore, India; 18 Enteric Diseases Group Murdoch Children’s Research Institute, Department of Paediatrics University of Melbourne, Parkville, Victoria, Australia; 19 National Institute for Viral Disease Control and Prevention, China CDC, Beijing, China; 20 Division of Viral Diseases, Bureau of Infectious Diseases Diagnosis Control, Korea Diseases Control and Prevention Agency, Osong, Korea; 21 Centre for Vaccine Innovation and Access, PATH, Geneva, Switzerland; 22 National Institute for Communicable Diseases, Centre for Enteric Disease, Johannesburg, South Africa; 23 Faculty of Health Sciences, Department of Medical Virology, University of Pretoria, Arcadia, Pretoria, South Africa; 24 Centers for Disease Control and Prevention, Atlanta, Georgia; 25 Retired Researcher, West Newton, Pennsylvania, United States of America; University of Michigan, UNITED STATES

## Abstract

Rotavirus is the most common pathogen causing pediatric diarrhea and an important cause of morbidity and mortality in low- and middle-income countries. Previous evidence suggests that the introduction of rotavirus vaccines in national immunization schedules resulted in dramatic declines in disease burden but may also be changing the rotavirus genetic landscape and driving the emergence of new genotypes. We report genotype data of more than 16,000 rotavirus isolates from 40 countries participating in the Global Rotavirus Surveillance Network. Data from a convenience sample of children under five years of age hospitalized with acute watery diarrhea who tested positive for rotavirus were included. Country results were weighted by their estimated rotavirus disease burden to estimate regional genotype distributions. Globally, the most frequent genotypes identified after weighting were G1P[8] (31%), G1P[6] (8%) and G3P[8] (8%). Genotypes varied across WHO Regions and between countries that had and had not introduced rotavirus vaccine. G1P[8] was less frequent among African (36 vs 20%) and European (33 vs 8%) countries that had introduced rotavirus vaccines as compared to countries that had not introduced. Our results describe differences in the distribution of the most common rotavirus genotypes in children with diarrhea in low- and middle-income countries. G1P[8] was less frequent in countries that had introduced the rotavirus vaccine while different strains are emerging or re-emerging in different regions.

## Background

Diarrheal diseases account for about ten percent of deaths in children under five years of age globally, with most of the burden occurring in low- and middle-income countries [1]. The leading cause of diarrheal diseases in children under five is rotavirus, although the introduction of rotavirus vaccines in the routine immunization schedules of more than one hundred countries has led to a significant decrease in the proportion of pediatric diarrheal cases and deaths attributable to rotavirus globally [2–5]. Despite this public health success, an estimated 128,500 deaths in children under five remained attributable to rotavirus in 2016, for the vast majority in low- and middle-income countries [3].

Group A rotaviruses are the most common rotaviruses in humans. Traditionally, they are classified into P (for protease-sensitive) and G (for glycoprotein) types based on immunological reactivities (serotypes) and gene sequences of VP4 and VP7 (genotypes), the two surface proteins expressed by rotaviruses [6,7]. Rotavirus genomes evolve through accumulation of point mutations but can also undergo genetic reassortment because their genome is composed of 11 double-stranded RNA segments. Such genetic changes, including those occurring between vaccine and wild-type strains, could lead to the appearance of new virus strains [8]. The emergence of these strains could in turn affect the impact and effectiveness of rotavirus vaccines.

Four vaccines are currently prequalified by WHO. Rotarix® (GlaxoSmithKline) is a monovalent live-attenuated vaccine based on a single G1P[8] strain. RotaTeq® (Merck) is a pentavalent human-bovine reassortant vaccine with G1, G2, G3, G4 and P[8] human rotavirus antigens [9]. These two vaccines were prequalified between 2008 and 2009 and are the most widely used rotavirus vaccines worldwide (including during the period covered by this study). Additional to homotypic protection, previous studies suggested that these two vaccines may also offer protection against other common and less common genotypes [10]. Two additional vaccines were prequalified by WHO in 2018: Rotavac® (Bharat Biotech) and Rotasiil® (Serum Institute of India). Both vaccines were used in some regions of India prior to WHO prequalification.

The objectives of this ecological analysis are to describe rotavirus genotypes circulating in low- and middle-income countries from different regions of the world as well as differences observed between countries that introduced the rotavirus vaccine in their national immunization schedule and countries that did not introduce the vaccine.

## Methods

### Data source

We extracted genotyping results from surveillance data reported to WHO by sentinel hospitals across all six WHO Regions participating in the Global Rotavirus Surveillance Network (GRSN) between 2014 and 2018. Established in 2009, this WHO-coordinated surveillance network aims to provide countries with evidence to support the introduction of rotavirus vaccines, monitor the impact of vaccine introduction and to identify circulating genotypes pre- and post-vaccine introduction [11]. The GRSN is supported by a network of laboratories that perform case confirmation and molecular characterization of rotavirus strains using validated standard protocols [12]. Surveillance activities that are part of the GRSN are exempt from ethical review as they are considered public health surveillance.

### Case definition in GRSN

Participating hospitals enrolled children under five years of age hospitalized with acute gastroenteritis. Acute gastroenteritis is defined as three or more episodes of loose stools in a 24h period. Cases presenting with blood in stools or with diarrhea lasting more than 14 days were considered inconsistent with the suspect case definition for rotavirus and excluded from surveillance. Rotavirus positivity was assessed using an enzyme-linked immunosorbent assay (ELISA) for detection of rotavirus antigen in fecal specimens following standard WHO protocols [12].

### Genotyping

Sentinel hospitals randomly selected 50 rotavirus-positive stool samples to be referred to a National or Regional Reference Laboratory for genotyping each year. However, some hospitals did not enroll enough positive cases to reach this threshold, in which case they sent all positive cases for genotyping. Some countries also chose to genotype a larger number of specimens, such as during vaccine effectiveness studies. The number of specimens genotyped may thus vary significantly between countries.

Identification of G and P genotypes was conducted by direct extraction of RNA from fecal specimens followed by reverse transcription-polymerase chain reaction (RT-PCR) methods and/or genetic sequencing by National Reference Laboratories (where genotyping/sequencing capacity is available) or by one of the 8 Regional Reference Laboratories of the GRSN (S1 Table) [12,13]. An informal group of technical experts from WHO and the global and regional reference laboratories recommended that at least 10% of samples genotyped using RT-PCR be sequenced and the genotype determined by genetic and phylogenetic analyses of sequence data, if sequencing capacities allows. This includes sequencing all non-typeable specimens by PCR and the unusual G-P combinations.

### Data analysis

We restricted all analyses to countries that reported at least 50 genotypes for the period 2014–2018, as recommended by an internal group of experts. Prior to 2014, genotyping results were reported to WHO in an aggregated format which only included the most common genotypes circulating at the time, making long term comparisons difficult. Data from more recent years were more sparse or not available at the time of this analysis.

We estimated the genotype frequency distribution for each of the 6 WHO Regions by aggregating country-level genotype distributions weighted by the level of rotavirus disease burden in each country. Frequency weighting was done as follows. First, we estimated the annual number of rotavirus infections in each GRSN country by multiplying the estimated number of pediatric diarrhea hospitalizations for 2017 [14] by the rotavirus positivity rate among pediatric diarrhea cases observed in each country for the period 2014–2018 in GRSN. We then multiplied these estimates by the unweighted genotype frequency distribution of each identified genotype in GRSN to estimate the number of rotavirus cases by genotype and country. Although non-typeable samples could represent new or unusual strains not included in the primers used to genotype a sample, they could also be caused by problems with the sample or the genotyping process itself. Large proportions of these non-typeable samples in high disease burden countries could significantly affect the Regional estimates computed in this study. They were thus excluded from this analysis prior to weighing and reported separately as raw numbers. Country-level results were then aggregated by WHO Region to derive the regional and global genotype frequency distributions. Genotypes reported in our results are those that represented at least 1% of the global or any regional distributions. We also estimated regional genotype distributions in the African and European Regions for countries that had introduced or not introduced the rotavirus vaccine in their routine immunization schedule separately. This was not possible in other WHO Regions that only included data from countries in the pre- or post-introduction eras. Information on year of vaccine introduction and brand of vaccine used in each country was extracted from WHO/UNICEF Joint Reporting Form (JRF) database [15]. Rotavirus vaccine was considered to be introduced in a country for a specific year if it was included in the national immunization program (for the entire country) as of 31 December of the previous year.

## Results

**Fig 1:**
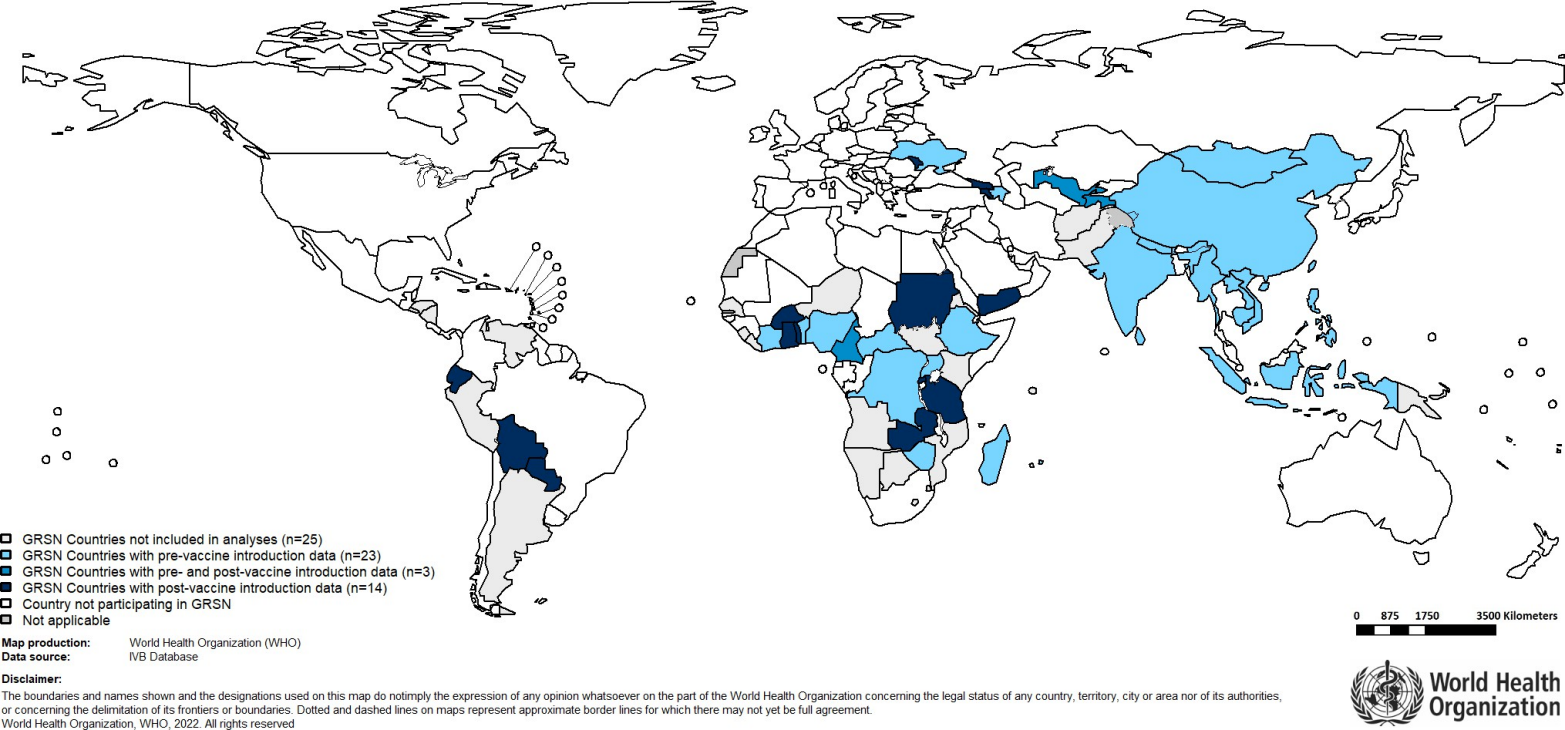
Countries included in this analysis (2014–2018).

A total of 224,060 children under the age of five hospitalized for acute watery diarrhea were enrolled over the period 2014–2018 in 65 countries across the six WHO Regions. Of those, 53,717 (24%) tested positive for rotavirus and 17,626 (33%) of positive samples were genotyped, across 56 countries. Sixteen countries did not meet the minimum number of specimens genotyped to be included in the analysis (50 samples) and were excluded. The final dataset included 16,967 specimens from 40 countries, of which 16,216 (96%) could be genotyped. Most countries (36) included only data either pre- (23 countries) or post- (14 countries) rotavirus vaccine introduction while Cameroon, Tajikistan and Uzbekistan contributed data to both pre- and post- vaccine periods. Fig 1 presents the countries included in the analysis and whether they provided pre-, and/or post- vaccine introduction data. The list of countries is also available in S2 Table, together with the years of data included for each country, the year of vaccine introduction in the national immunization schedule and the vaccine product used.

**Fig 2:**
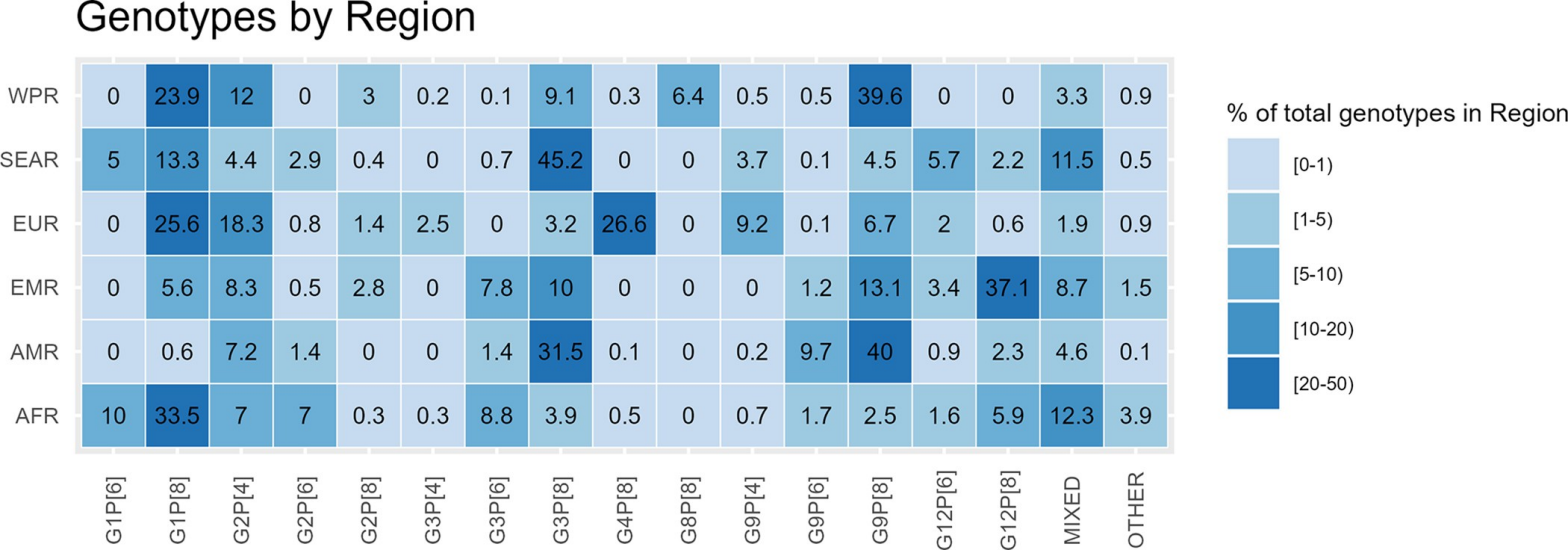
Distribution of the most common rotavirus A genotypes by World Health Organization Region, weighted (2014–2018). AFR: African Region, AMR: Region of the Americas, EMR: Eastern Mediterranean Region, EUR: European Region, SEAR: South-East Asia Region, WPR: Western Pacific Region.

We identified a total of 15 genotypes which comprised at least 1% of the global or at least one regional distribution after weighting. Globally, the most frequent genotypes identified were G1P[8] (31%), G1P[6] (8%) and G3P[8] (8%). Eleven percent of samples were of mixed G, P or both G and P types (Fig 2, Table 1).

**Table 1:** Genotype distribution by World Health Organization Region, 2014–2018.

	AFR	AMR	EMR	EUR	SEAR	WPR	TOTAL
**Countries (n)**	17	3	2	7	5	6	40
**Samples typed**	3036	395	321	2726	1218	8520	16216
**Samples Non typeable**	119	79	21	65	141	326	751
**Genotypes (%)**							
G1P[8]	33.54	0.64	5.55	25.63	13.26	23.94	30.55
G1P[6]	9.97	0.00	0.00	0.00	5.04	0.04	8.45
G3P[8]	3.94	31.47	10.01	3.24	45.21	9.12	8.20
G2P[4]	7.03	7.24	8.29	18.34	4.36	12.03	7.33
G3P[6]	8.79	1.42	7.79	0.00	0.71	0.05	7.17
G9P[8]	2.50	40.01	13.08	6.74	4.50	39.63	6.43
G2P[6]	6.99	1.45	0.52	0.77	2.88	0.02	5.88
G12P[8]	5.95	2.27	37.06	0.58	2.15	0.02	5.18
G12P[6]	1.56	0.93	3.38	1.98	5.74	0.00	1.79
G9P[6]	1.68	9.66	1.24	0.13	0.09	0.54	1.44
G9P[4]	0.69	0.19	0.00	9.24	3.67	0.45	0.96
G8P[8]	0.04	0.00	0.00	0.00	0.00	6.44	0.66
G2P[8]	0.35	0.00	2.83	1.39	0.36	3.00	0.62
G4P[8]	0.51	0.08	0.00	26.64	0.00	0.34	0.55
G3P[4]	0.25	0.00	0.00	2.50	0.04	0.18	0.23
**MIXED**	12.29	4.56	8.75	1.88	11.52	3.34	11.28
**OTHER**	3.03	0.08	1.50	0.92	0.47	0.87	1.93

AFR: African Region, AMR: Region of the Americas, EMR: Eastern Mediterranean Region, EUR: European Region, SEAR: South East Asia Region, WPR: Western Pacific Region.

The most common genotypes, defined by those comprising >10% of cases, differed by WHO Region. In the African Region, the most common genotype was G1P[8] (34%), followed by G1P[6] (10%). In the Region of the Americas, G9P[8] was the most common genotype (40%) followed by G3P[8] (31%). In the Eastern Mediterranean Region, G12P[8] was the most common (37%), followed by G9P[8] (13%) and G3P[8] (10%). In the European Region, G4P[8] was the most frequent genotype (27%), followed by G1P[8] (26%) and G2P[4] (18%). In the South East Asia Region, G3P[8] was the most common genotype (45%), followed by G1P[8] (13%). In the Western Pacific Region, G9P[8] represented 40% of all genotypes followed by G1P[8] (24%) and G2P[4] (12%) (Fig 2, Table 1).

In the African Region, G1P[8] (36%) was the most common genotype in countries that had not introduced the rotavirus vaccine, followed by G1P[6] (11%). In countries that had already introduced the vaccine, G1P[8] (20%) and G2P[4] (14%) were the most common circulating genotypes post-vaccine introduction.

In the European Region, G1P[8] (33%), G4P[8] (25%), and G2P[4] (12%) were the most frequent genotypes in countries that had not introduced rotavirus vaccine. G2P[4] was the most frequent genotype (28%), followed by G9P[4] (18%) and G4P[8] (17%) in countries that had introduced the vaccine, while G1P[8] represented only 8% of all genotyped cases in these countries. (Table 2).

**Table 2:** Genotype distribution in the African Region, and European Region by country pre- or post-vaccine-introduction status, 2014–2018.

	AFR		EUR	
	Pre-	Post	Pre-	Post
**Countries (N)**	11	8	4	5
**Genotypes (N)**	1850	1186	1269	1457
**Non typeable (N)**	103	16	20	45
**Genotypes (%)**				
G1P[6]	10.62	5.48	0	0
G1P[8]	36.4	20.08	32.65	8.24
G2P[4]	5.61	13.59	12.44	27.75
G2P[6]	7.24	6.63	0	2.75
G2P[8]	0	0.63	0	2.47
G3P[4]	0	1.13	0	9.55
G3P[6]	8.85	11.15	0	0
G3P[8]	3.42	5.92	3.54	2.3
G4P[6]	0.32	1.4	1.71	0.33
G4P[8]	0.19	1.95	24.99	16.79
G9P[4]	0.62	0.97	12.2	17.73
G9P[6]	1.84	0	0.13	0
G9P[8]	2.75	0.82	5.31	9.14
G12P[6]	0.92	4.33	3.24	1.22
G12P[8]	5.38	7.52	0.8	0.04
**MIXED**	12.26	10.92	1.91	1.62
**OTHER**	3.58	7.49	1.1	0.06

AFR: African Region, EUR: European Region.

## Discussion

Our results provide unique insights on the distribution of rotavirus genotypes circulating in low- and middle-income countries across all WHO Regions. These results are based on more than 16,000 genotypes identified between 2014 and 2018 in 40 countries participating in GRSN. This is by far the largest and geographically diverse compilation of original rotavirus strain surveillance data from low- and middle-income countries to date, which have been underrepresented in published and available rotavirus genotype data, despite having the greatest burden of rotavirus disease. Globally, G1P[8] was by far the most common genotype (31%) while all other genotypes represented less than 10% of all identified strains. However, important variations were observed across WHO Regions and between countries that had introduced the rotavirus vaccine and those that had not.

G1P[8] was the most frequent genotype in the African Region and the second most frequent in the European Region. In both Regions, the proportion of G1P[8] genotypes in countries that had introduced the rotavirus vaccine was much lower than in countries that were yet to introduce the vaccine. This difference was particularly marked in the European Region (33% vs 8%). In the Americas where the rotavirus vaccine has been introduced in most countries for longer (since 2006), G1P[8] genotype was almost completely absent (<1%). A low proportion of this genotype was also observed in the Eastern Mediterranean Region (6%) which included two countries that had already introduced the vaccine in their immunization schedule. This is in line with previous studies that reported a replacement of the predominant genotype in the pre-vaccine era (G1P[8]) by other genotypes post- vaccine introduction, together with a broader heterogeneity in genotype distribution [16,17]. Our results from the African and European Regions support previous evidence of the efficacy of the Rotarix® and Rotateq® vaccines against the G1P[8] genotype [10].

G3P[8], G9P[8], and G12P[8] were frequently identified in the past but were overall quite rare in the pre-vaccine introduction years. Previous studies reported that these genotypes increased in proportion following rotavirus vaccine introduction [8,16–22]. In this study, G9P[8] and G3P[8] accounted for more than 70% of all genotypes identified in the Americas where rotavirus vaccine use is common, supporting previous evidence [21]. However, G3P[8] and G9P[8] also accounted for 40% or more of genotypes identified in the South East Asia and Western Pacific Regions, respectively. These two Regions included only countries that had not introduced the rotavirus vaccine nationally. Similarly, in Thailand where the rotavirus vaccine has yet to be introduced nation-wide, G3P[8] was the most frequent genotype between 2016 and 2019 [23]. In Australian States using the RotaTeq® vaccine, G12P[8] was previously reported as the dominant genotype post-introduction but in contrast this was not observed in States using Rotarix® [17]. In Italy (Rotarix®) and Cote d’Ivoire (RotaTeq®), G12P[8] was also the most frequent genotype reported pre-introduction [19,24]. In our study, G12P[8] was by far the most common genotype in the Eastern Mediterranean Region which included two countries that had introduced the Rotarix® vaccine. G12P[8] remained quite rare in other Regions. In the African Region, the frequencies of the G3P[8], G9P[8] and G12P[8] genotypes were not different in countries that had introduced the vaccine compared with those countries that had not.

These findings suggest that the G3P[8], G9P[8] and G12P[8] genotypes are circulating globally, in varying frequencies and regardless of vaccine use. It is however also possible that the high proportion of these genotypes in countries that are yet to introduce the vaccine could reflect transmission from a neighboring region or country where the rotavirus vaccine is used.

In this analysis, G2P[4] represented about 4 to 12% of genotypes in most Regions. However, it was the third most frequent genotype in the European Region with a proportion twice as high in countries that had introduced the vaccine (all using Rotarix®) as compared to countries that had not introduced. A similar pattern was observed in the African Region where G2P[4] was the second most common genotype in countries that had introduced the vaccine. G2P[4] was previously reported as the most commonly identified genotype in England post-vaccine introduction [16]. It was also the most common strain identified in European countries with Rotarix® in their national immunization schedule and participating in the EuroRotaNet project [25]. In contrast, this was not observed in Finland where RotaTeq® is used [22]. In Australia, G2P[4] was also reported to be frequent post-introduction, more so in states using the Rotarix® vaccine than in those using RotaTeq® [17]. These data could suggest that immune pressure from Rotarix vaccine causes selection for genotype G2P[4] strains. One can hypothesize that in pre-vaccine settings, the natural fluctuation of genotypes induces a broad immune protection in the community, whereas a vaccine tends to skew immune pressure in a single direction with a rise in prevalence of specific genotypes. It will be interesting to monitor this trend as additional countries introduce rotavirus vaccination to support or infirm such theory.

G4P[8] was one of the most common genotypes in the European Region (in both pre- and post-vaccine introduction countries) but was almost completely absent from all other Regions. G4P[8] was previously found to be rare in Australia post-vaccine introduction as opposed to the pre-vaccine era [17].

G1P[6], G2P[6] and G3P[6] were found in the African Region but were absent from most other Regions.

This is consistent with previous reports of the high prevalence of P[6] strains in symptomatic children from the African continent [26]. G3P[4] and G9P[4] were found in the European Region and in the case of G3P[4], only in countries that had introduced the rotavirus vaccine. Similar strain emergence was previously reported in Ghana post vaccine introduction [20].

This analysis of GRSN rotavirus genotype data has a number of limitations. The ecological design limits the interpretation of the effect of vaccine introduction on genotype distribution since vaccine introduction at the country-level may not reflect the vaccination status of individuals from that country. Different vaccine products used by countries within each WHO Region and effective against different strains of rotaviruses could also alter our results, although most countries participating in GRSN are using Rotarix® which limits this issue (S1 Table). Differences in genotype distributions could be due to geography and/or natural fluctuations rather than vaccine introduction as WHO Regions span very wide geographic areas and include countries with very different population profiles.

The regional estimates presented in this paper only include countries participating in the GRSN, though these countries represent a large proportion of low- and middle-income countries globally. The representativeness of these regional estimates could also be altered by the limited number of country-years of data available, particularly in the Regions of the Americas and the Eastern Mediterranean. Although not a limitation per se, regional estimates are driven by results from countries with high rotavirus disease burden and are thus not representative of all countries in the Region. Because of the limited catchment area of the sentinel surveillance hospitals participating in the GRSN, and the limited number of samples per country, the genotype distributions observed in hospitals may not reflect the genotype distributions of the entire country.

Because the data were aggregated over a 5-year surveillance period we are not able to report changes within that period. GRSN enrolls only severe, hospitalized children and although studies from the pre-vaccine era did not identify clear associations between genotype and disease severity, milder disease presentations may be associated with strains for which vaccines protect against severe forms of disease more efficiently. Including only severe cases could thus distort the true strain distribution.

The main strength of this study is the large number of rotavirus strains included and the large number of countries from all WHO Regions included in the analyses. GRSN has been established for more than 10 years and uses a standardized surveillance and laboratory protocols. Thanks to these standardized protocols, samples genotyped are likely to be representative of the entire surveilled cohort. The performance of sentinel hospitals participating in GRSN is regularly evaluated and reviewed through WHO quality assurance programs. Laboratories supporting diagnosis confirmation and/or genotyping within GRSN undergo quality control exercises annually with very high levels of performance [27,28]. Finally, the weighting process applied ensures that each country is represented adequately within regional and global estimates.

In conclusion, this unique ecological study provides broadly representative data on regional and global rotavirus genotype distribution in low- and middle- income countries. It reports low prevalence of G1P[8] in the post-vaccine introduction era broadly across regions. It also highlights the geographical differences observed in circulating genotypes pre- and post- vaccine introduction. This suggests that the emergence or re-emergence of new genotypes post-introduction may not be attributed directly to vaccine use. However, our findings can potentially encourage further analyses to be done at the country-level; longer surveillance time series can be done to disentangle natural temporal variations and to assess the impact of vaccine introduction or type of product used. These changes in circulating rotavirus genotypes do not seem to have diminished the overall impact of rotavirus vaccination globally yet; however, these changes stress the importance to continuing and sustaining rotavirus surveillance programs at the country-level, particularly post-vaccine introduction.

## Supporting information

S1 Table Global Rotavirus Surveillance Network (GRSN)—Global and Regional Reference Laboratories (2014–2018). (DOCX)

S2 Table Countries included and year of rotavirus a vaccine introduction in the entire country. (DOCX)
