# Supplementary material for: Rotavirus genotypes in children under five years hospitalized with diarrhea in low and middle-income countries: Results from the WHO-coordinated Global Rotavirus Surveillance Network
Source: PLOS Glob Public Health. 2023 Nov 28;3(11):e0001358. doi: 10.1371/journal.pgph.0001358 (PMC10683987; doi:10.1371/journal.pgph.0001358)
Supplement: S1 Table — (DOCX) [file pgph.0001358.s001.docx]

**S1 Table.** Global Rotavirus Surveillance Network (GRSN) - Global and Regional Reference Laboratories (2014-2018)

| **Region** | **Regional Reference Laboratory** |
| --- | --- |
| AFR | Noguchi Memorial Institute for Medical Research, Accra, Ghana |
| AFR | Sefako Makgatho Health Sciences University, Pretoria, South Africa |
| AMR | Oswaldo Cruz Institute, Fiocruz, Rio de Janeiro, Brasil |
| AMR | The US Centers for Diseases Control and Prevention (CDC), Atlanta, United States |
| EMR | Namru-3, Cairo (no longer in activity) |
| EUR | Republican Research and Practical Center for Epidemiology and Microbiology (RRPCEM), Minsk, Belarus |
| SEAR | Christian Medical College, Vellore, India |
| WPR | Murdoch’s Children Research Institute, Melbourne, Australia |
| WPR | Chinese Center for Disease Control and Prevention, Beijing, China |
| WPR | Korea Centers for Disease Control and Prevention, Osong, South Korea |
