# Supplementary material for: Rotavirus genotypes in children under five years hospitalized with diarrhea in low and middle-income countries: Results from the WHO-coordinated Global Rotavirus Surveillance Network
Source: PLOS Glob Public Health. 2023 Nov 28;3(11):e0001358. doi: 10.1371/journal.pgph.0001358 (PMC10683987; doi:10.1371/journal.pgph.0001358)
Supplement: S2 Table — (DOCX) [file pgph.0001358.s002.docx]

**S2 Table.** Countries included and year of rotavirus A vaccine introduction in the entire country

| WHO Region | Country | Specimens with rotavirus genotypes identified | Years of surveillance included in analysis | Year of rotavirus Vaccine introduction | Rotavirus Vaccine product used |
| --- | --- | --- | --- | --- | --- |
| AFR | Benin | 93 | 2014, 2016, 2018 |  |  |
| AFR | Burkina Faso | 260 | 2014, 2015, 2018 | 2013 | RotaTeq |
| AFR | Cameroon | 261 | 2014, 2015, 2016 | 2014 | Rotarix |
| AFR | Central African Republic | 165 | 2014, 2016, 2017, 2018 |  |  |
| AFR | Cote d'Ivoire | 81 | 2014, 2015, 2016, 2017 | 2017 | RotaTeq |
| AFR | Democratic Republic of the Congo | 78 | 2015, 2016 |  |  |
| AFR | Ethiopia | 51 | 2014 | 2014 | Rotarix |
| AFR | Ghana | 191 | 2014, 2016, 2018 | 2012 | Rotarix |
| AFR | Madagascar | 122 | 2014 | 2014 | Rotarix |
| AFR | Mauritius | 67 | 2014 | 2015 | Rotarix |
| AFR | Nigeria | 742 | 2014, 2015, 2016, 2018 |  |  |
| AFR | Rwanda | 68 | 2014 | 2012 | Rotarix |
| AFR | Togo | 112 | 2015, 2016, 2017, 2018 | 2014 | Rotarix |
| AFR | Uganda | 175 | 2018 | 2018 |  |
| AFR | United Republic of Tanzania | 201 | 2014, 2018 | 2013 | Rotarix |
| AFR | Zambia | 184 | 2014, 2017, 2018 | 2013 | Rotarix |
| AFR | Zimbabwe | 185 | 2014 | 2014 |  |
| AMR | Bolivia (Plurinational State of) | 135 | 2018 | 2008 | Rotarix |
| AMR | Ecuador | 131 | 2016, 2017, 2018 | 2007 | Rotarix |
| AMR | Paraguay | 129 | 2014, 2015, 2016, 2017, 2018 | 2010 | Rotarix |
| EMR | Sudan | 239 | 2014, 2015 | 2011 | Rotarix |
| EMR | Yemen | 82 | 2014 | 2012 | Rotarix |
| EUR | Armenia | 357 | 2014, 2015, 2016, 2017, 2018 | 2012 | Rotarix |
| EUR | Azerbaijan | 287 | 2014, 2015, 2016, 2017, 2018 |  |  |
| EUR | Georgia | 264 | 2014, 2015, 2016, 2017, 2018 | 2013 | Rotarix |
| EUR | Republic of Moldova | 372 | 2014, 2015, 2016, 2017, 2018 | 2012 | Rotarix |
| EUR | Tajikistan | 408 | 2014, 2015, 2016, 2017, 2018 | 2015 | Rotarix |
| EUR | Ukraine | 723 | 2014, 2015, 2016, 2017, 2018 |  |  |
| EUR | Uzbekistan | 315 | 2014, 2015, 2016, 2017, 2018 | 2014 | Rotarix |
| SEAR | India | 250 | 2017, 2018 |  |  |
| SEAR | Indonesia | 107 | 2014, 2015 |  |  |
| SEAR | Myanmar | 440 | 2014, 2015, 2016, 2017, 2018 |  |  |
| SEAR | Nepal | 340 | 2014, 2015, 2016, 2017 |  |  |
| SEAR | Sri Lanka | 81 | 2014, 2015 |  |  |
| WPR | Cambodia | 647 | 2014, 2015, 2016 |  |  |
| WPR | China | 1289 | 2014, 2015, 2016, 2017, 2018 |  |  |
| WPR | Lao People's Democratic Republic | 325 | 2014, 2015, 2016, 2017 |  |  |
| WPR | Mongolia | 359 | 2014, 2016 |  |  |
| WPR | Philippines | 4036 | 2014, 2015, 2016, 2017, 2018 |  |  |
| WPR | Viet Nam | 1864 | 2014, 2015, 2016, 2017, 2018 |  |  |

AFR: African Region, AMR: Region of the Americas, EMR: Eastern Mediterranean Region, EUR: European Region, SEAR: South East Asia Region, WPR: Western Pacific Region
